# Application progress of artificial intelligence in managing thyroid disease

**DOI:** 10.3389/fendo.2025.1578455

**Published:** 2025-06-17

**Authors:** Qing Lu, Yu Wu, Jing Chang, Li Zhang, Qing Lv, Hui Sun

**Affiliations:** ^1^ Department of Ultrasound, Union Hospital, Tongji Medical College, Huazhong University of Science and Technology, Wuhan, China; ^2^ Department of Endocrinology, Union Hospital, Tongji Medical College, Huazhong University of Science and Technology, Wuhan, China

**Keywords:** artificial intelligence, deep learning, thyroid nodule, ultrasonography, radiomics, pathology

## Abstract

Artificial intelligence (AI) has been used to study thyroid diseases since the 1990s. Previously, it mainly concentrated on the diagnosis of thyroid function and distinguishing benign from malignant thyroid nodules. With the rapid development of machine and deep learning, AI has been widely used in multiple areas of thyroid disease management, including image analysis, pathological diagnosis, personalized treatment, patient monitoring, and follow-up. This review systematically examines the evolution of AI applications in thyroid disease management since the 1990s, with a focus on diagnostic innovations, therapeutic personalization, and emerging challenges in clinical implementation. AI not only reduces the subjectivity associated with ultrasound examinations but also enhances the differentiation rate of benign and malignant thyroid nodules, thereby reducing the frequency of unnecessary fine-needle aspirations. AI synthesizes multimodal data, such as ultrasound, electronic health records, and wearable sensors, for continuous health monitoring. This integration facilitates the early detection of subclinical recurrence risk, particularly in patients who have undergone thyroidectomy. Despite the broad prospects of AI applications, challenges related to data privacy, model interpretability, and clinical applicability remain. This review critically evaluates studies across the ultrasound, CT/MRI, and histopathology domains, while addressing barriers to clinical translation, such as data heterogeneity and ethical concerns.

## 1 Introduction

The origins of artificial intelligence (AI) can be traced back to the 1950s, when researchers first sought to simulate human thought and decision-making processes (1). With the rapid advancement of computer technology, AI applications have expanded, notably in medical image analysis, where AI has been integrated into computer-aided diagnosis systems to detect and evaluate abnormal structures (2). In the context of thyroid disease research, which began in the 1990s, early AI applications primarily focused on assessing thyroid function (3) and analyzing ultrasound images to assist clinicians in differentiating between benign and malignant nodules (4).

Thyroid nodules are common in the general population. Approximately 20% of individuals have palpable thyroid nodules on physical examination, and up to 50% present with nodules on imaging. However, only 5% to 15% of these cases are malignant (5). Fine-needle aspiration (FNA) biopsy is the gold standard for the preoperative diagnosis of thyroid cancer. Current diagnostic methods detect 20%–30% of cytologically indeterminate thyroid nodules, with a false-negative rate of 3% to 5%, depending on cytological interpretation and nodule characteristics (6, 7). Currently, the major clinical challenge in the management of thyroid nodules is the diagnosis of thyroid cancer. A large multicenter correlation study found a 34% malignancy rate for FNAs with indeterminate cytology (8). However, the American College of Radiology Thyroid Imaging Reporting and Data System (ACR TI-RADS) risk stratification system is relatively complex to apply in clinical practice and has limited diagnostic specificity (44%-67.3%) (9). Clinicians require additional tools to reduce overdiagnosis and avoid unnecessary surgeries.

In the 21st century, rapid advancements in machine and deep learning have created transformative opportunities for AI applications in thyroid disease management. The latest deep-learning algorithms have markedly enhanced image-processing capabilities, allowing AI to analyze complex ultrasound images with greater accuracy and thereby improve diagnostic sensitivity and specificity (10). For instance, studies indicate that AI-assisted ultrasound diagnostic systems can achieve accuracy rates exceeding 90% for identifying thyroid nodules, significantly surpassing traditional diagnostic methods (11). AI combined with radiomics can reduce the rate of unnecessary FNA biopsies from 30.0% to 4.5% in the validation dataset and from 37.7% to 4.7% in the test dataset, compared to ACR TI-RADS (9). AI systems can identify subtle changes in cellular morphology and tissue structure (12, 13) improving the diagnostic accuracy of FNA biopsies (14, 15). In a comparison between AI and human experts, the AI model demonstrated higher accuracy and specificity than those of the average expert cytopathologist by more than two standard deviations (accuracy 99.71% *vs*. 88.91%, sensitivity 99.81% *vs*. 87.26%, and specificity 99.61% *vs*. 90.58%) (16).

**Figure 1:**
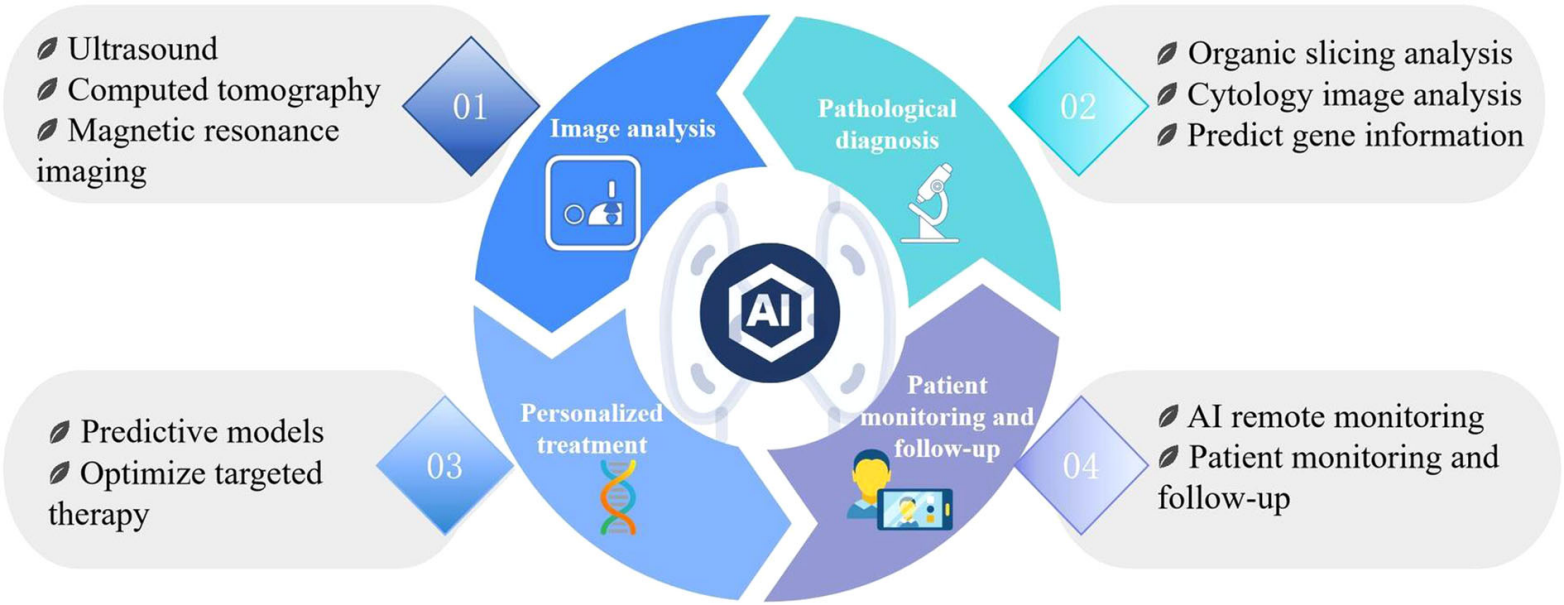
AI is extensively applied across various aspects of thyroid disease management, including image analysis, pathological diagnosis, personalized treatment, as well as patientmonitoring and follow-up.

Currently, AI is extensively applied to various aspects of thyroid disease management, including image analysis (17–20), pathological diagnosis (12, 14, 16, 21, 22), personalized treatment (23, 24), and patient monitoring and follow-up (25, 26) (**Figure 1**). By leveraging historical case data, AI systems offer considerable advantages in standardized diagnoses, risk assessments, personalized treatments, and patient follow-ups, ultimately providing more accessible and tailored healthcare services.

In summary, advancements in AI for thyroid disease management exemplify the deep integration of medicine and computer science, presenting significant opportunities to advance personalized healthcare. This study aims to review the current progress of AI applications in thyroid disease and explore future directions for its development.

## 2 Methods

### 2.1 Search strategy and inclusion criteria

**Figure 2:**
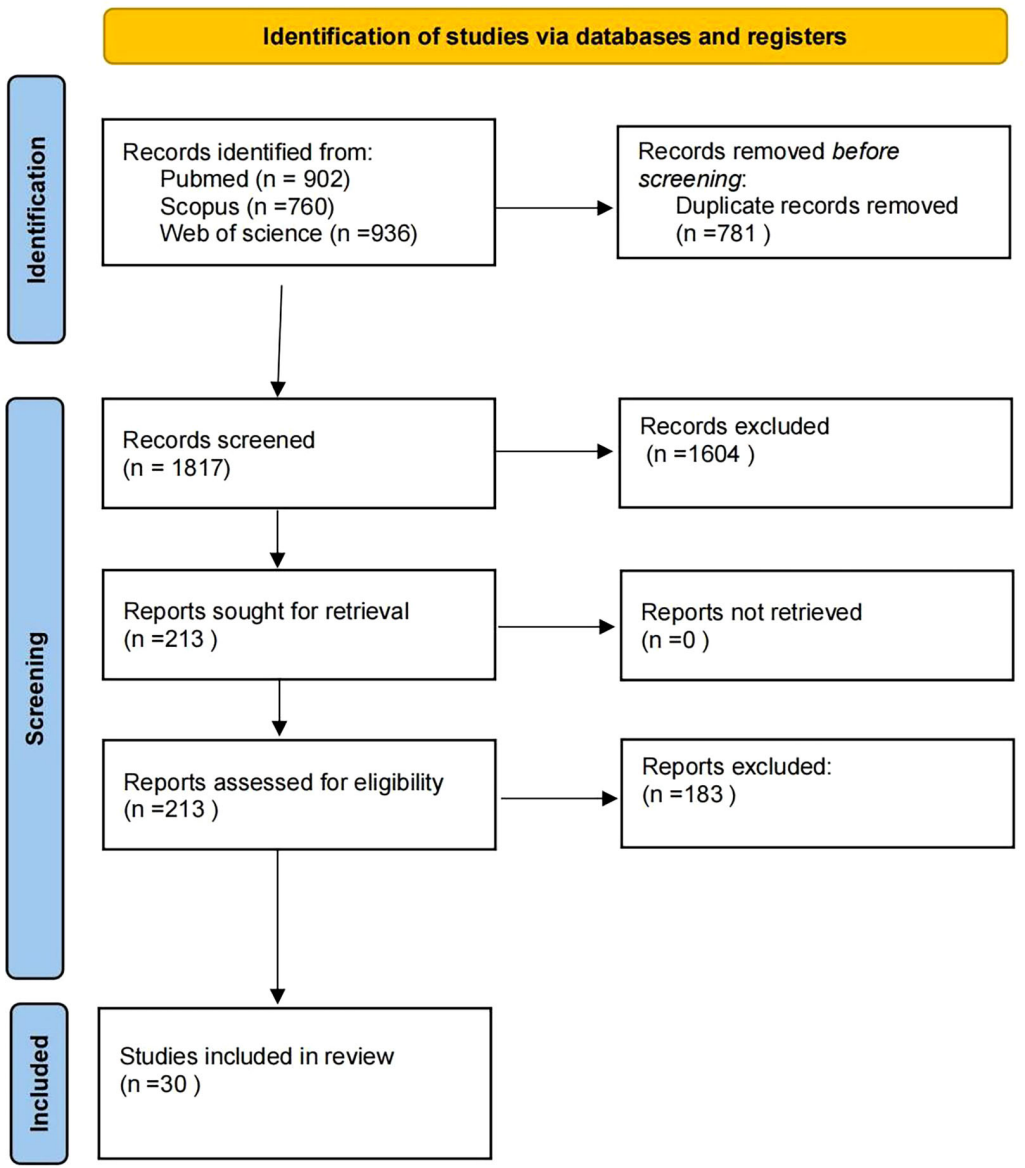
PRISMA flowchart of the review.

This review followed the PRISMA guidelines. Databases (PubMed, Scopus, and Web of Science) were searched (2019–2025) using the following keywords: ‘ultrasonography,’ ‘ultrasonics,’ ‘artificial intelligence,’ ‘intelligent learning,’ ‘thyroid nodule,’ ‘thyroid cancer,’ ‘pathology,’ ‘personalized treatment,’ ‘CT,’ and ‘MRI.’ Inclusion criteria: (1) Clinical human studies; (2) validation in ≥50 patients; (3) performance metrics reported. Exclusion criteria: (1) Animal or phantom studies; (2) technical reports without clinical validation. From 1,837 records, 30 studies met the criteria after screening (see the PRISMA flowchart, **Figure 2**).

### 2.2 Reproducibility

The reproducibility analysis revealed critical gaps: 90% (27/30) of the studies utilized proprietary datasets with restricted access, whereas 90% (27/30) failed to disclose preprocessing codes. A striking example of this “reproducibility crisis” is Peng’s ThyNet model (27), which achieved 89.1% accuracy in the original publication. However, independent replication attempts by Gild et al. demonstrated a performance decline to 64% (28). Standardized image storage protocols and preprocessing environments are urgently required to enhance reproducibility.

## 3 Results

### 3.1 Diagnostic applications

#### 3.1.1 Imaging analysis (Ultrasound/CT/MRI)

##### 3.1.1.1 Evolution of AI in ultrasound technology and clinical applications

###### 3.1.1.1.1 Early exploration of traditional AI algorithms

AI research on thyroid ultrasound originated in 1993 by Sharpe et al., who utilized artificial neural networks for *in vitro* thyroid function diagnosis (29). Early studies focused on constructing machine learning models based on ultrasound features manually extracted by radiologists, such as nodule morphology and echogenicity. For example, the thyroid ultrasound computer-aided diagnosis system developed by Choi et al. demonstrated a sensitivity comparable to that of experienced radiologists but exhibited lower specificity (56%) and overall accuracy (17). Similarly, the S-Detect system achieved 95% sensitivity for thyroid cancer diagnosis; however, its insufficient specificity (56%) highlights the risk of overdiagnosis (18). Although these technologies improve diagnostic consistency, they remain reliant on manual annotation or feature extraction, introducing subjectivity, operational complexity, and potential increases in interpretation time, false-positive rates, and false-negative rates (30).

###### 3.1.1.1.2 Revolution in autonomous feature extraction via deep learning

Deep learning, through multilayered neural networks enables automatic extraction of high-dimensional image features, overcoming the limitations of traditional methods. Examples include: the AIBx system developed by Swan et al., which integrated with TI-RADS classification and significantly reduced the risk of missed malignant nodules (single-center study, 413 nodules; AIBx and TI-RADS false-negative rates: 22% *vs*. 6%, with no malignant nodules overlooked when both methods concurred on benign classification) (19); the Al-Thyroid model designed by Eun et al., which improved diagnostic accuracy and interobserver consistency, particularly enhancing junior physicians’ performance (AUROC increased from 0.854 to 0.945, sensitivity from 84.2% to 92.7%, specificity from 72.9% to 86.6%; P <.001 for all) (20); and the ThyNet system proposed by Peng et al., which integrated ultrasound images and video data from 23 hospitals (18,049 images) to optimize positive/negative predictive values and reduce unnecessary FNAs (FNAs decreased from 61.9% to 35.2%, while missed malignancies declined from 18.9% to 17.0%) (27). However, most studies rely entirely on hospital-confirmed histopathological data and lack representation of screening populations. Differences in disease prevalence across cohorts may distort the PPV/NPV metrics and compromise generalizability. Additionally, the exclusion of nondiagnostic scans and unresolved multinodular correlations from retrospective datasets introduces methodological bias. Largescale screening validation remains critical to address these translational gaps.

Deep learning not only achieves benign–malignant nodule classification (AUC: 0.90) (31)but also synergizes with radiomics to extract quantitative features (including shape, texture, and intensity) for refined clinical decision-making. Examples include metastasis prediction: Yu et al.’s radiomics model predicted lymph node metastasis in thyroid cancer with an AUC of 0.90 (n = 1,013) (31); genomic and prognostic analysis: ultrasound features correlated with tumor phenotypes or genetic mutations (n = 96) (32), while multimodal models localized primary cancer sites in metastatic lymph nodes (n = 280) (33); and treatment optimization: radiomics-clinical integrated models reduced unnecessary central lymph node dissections (34, 35) and assessed disease-free survival (36). Most AI validations depend on single-center retrospective data, and lack largescale, multicenter prospective validations.

###### 3.1.1.1.3 Clinical value of AI-TI-RADS

A retrospective analysis of 2,061 thyroid nodules (sampled via FNA or surgery) was used to develop the AI-TI-RADS classification model. Compared to the conventional ACR TI-RADS, AI-TI-RADS demonstrated superior specificity (70.2% *vs*. 49.2%) and biopsy avoidance rates (42.3%), while maintaining comparable sensitivity (82.2% *vs*. 86.7%) (37). This disparity underscores the need to balance sensitivity and specificity based on clinical scenarios (37).

##### 3.1.1.2 AI advancements in CT and MRI

Although ultrasound remains the primary imaging modality for thyroid disorders, CT is indispensable in complex cases, such as the assessment of tumor invasiveness. The AI system developed by Wang et al. predicted preoperative cervical lymph node metastasis in thyroid cancer using CT images and outperformed senior radiologists in sensitivity and accuracy. When combined with radiologists, AI further enhances diagnostic efficacy, demonstrating its utility in surgical planning (38). MRI, with its high soft tissue resolution, offers unique advantages for assessing extrathyroidal extension. A radiomics study (n = 132) identified 16 key features from multiparametric MRI data, constructing a predictive model for extrathyroidal extension with an AUC of 0.87 (39). However, these studies involved moderate sample sizes, necessitating larger cohorts to improve predictive efficiency. Additionally, deep learning-based segmentation of thyroid lesions on CT or MRI remains unexplored in the literature. Tumors <0.5 cm in diameter were excluded because of unreliable identification and segmentation on CT or MRI images.

#### 3.1.2 Pathology support

The earliest applications of AI in the pathological analysis of thyroid diseases date back to the 1990s, when AI was primarily used for basic image recognition and classification. Researchers began exploring computer-assisted techniques for analyzing pathological slides; however, the limitations of the technology restricted its application (40).

In the 21st century, the rapid development of deep learning has significantly advanced the application of AI in pathological analysis. The introduction of convolutional neural networks has enabled AI to effectively process and analyze high-resolution pathological images. Research during this period has focused on automated tumor detection and classification, particularly in the diagnosis of thyroid cancer, where AI systems can identify subtle changes in cellular morphology and tissue structure (12, 13). Guan et al. utilized a the VGG-16 deep convolutional neural network (DCNN) model to establish a pathology-validated dataset from 279 cytological images of thyroid nodules. They trained and tested both the VGG-16 and Inception-v3 DCNN models and found that the VGG-16 model showed significant potential to enhance the diagnosis of papillary thyroid carcinoma (PTC) in cytological images. In fragment images, the contours, perimeter, area, and average pixel intensity of PTC cells were all greater than those of benign nodules (12). FNA biopsy remains the gold standard for the preoperative diagnosis of malignant tumors. However, approximately 10%–30% of thyroid nodules yield inconclusive results, with 10-40% of those cases subsequently confirmed to be malignant (41). Zhao et al., found that the DCNN ResNeSt achieved high sensitivity in diagnosing malignancies in these ambiguous atypical nodules. The ResNeSt model achieved an accuracy of 92.49% (160/173) on fragment images and 84.78% (39/46) in distinguishing PTC from benign nodules in ambiguous cases. The sensitivity and specificity of the ResNeSt model were 95.79% and 88.46%, respectively. Malignant nodules exhibit larger and more deeply stained nuclei than those of benign nodules (14).

The development of AI-assisted algorithms using digital cytology images has been significantly impeded by technical challenges and a shortage of optimized scanners for cytology specimens (42). In a study by Guan et al., all three fragmented false-positive cases showed large nuclei with high mean pixel color information similar to that of malignant cells. However, cytopathologists considered these images representative of typical benign nodules. The authors suggested that the DCNN based its diagnosis on nucleus size and staining intensity rather than shape.Future studies should focus on training the networks to differentiate between cellular and nucleus morphologies (12). Additionally, current DCNN models require sufficient sample sizes; smaller datasets risk overfitting. Rare thyroid cancer histopathologies—such as follicular thyroid cancer (FTC) and Hürthle cell carcinoma—remain difficult to diagnose. Wai-Kin Chan et al. found that the accuracy of convolutional neural networks in identifying FTC was only 63.6%–72.7% and in identifying Hürthle cell carcinoma, only 60%–66.7%. These limitations were largely because of the small number of cases in the database—a consequence of the low incidence and prevalence of these cancers. However, the performance of retrained convolutional neural networks was significantly better than that of the participating physicians (43).

Since 2016, the application of AI has gradually evolved toward the integration of multimodal data. Researchers have begun to explore combinations of pathological images with clinical data and genomic information to construct comprehensive models (44). This trend extends the capabilities of AI beyond image analysis, enabling support for predicting genetic information, assessing patient prognosis, and developing personalized treatment plans. For example, PTC— particularly its aggressive subtype—is often associated with BRAF p.V600E mutations and RET fusions (45, 46). Rossi et al. examined 72 FNA cytology specimens from patients diagnosed with PTC and found that 47 of the patients with mutations exhibited distinct morphological features. This study demonstrated that the BRAF p.V600E mutation could be predicted in cytological samples based on specific morphological characteristics (21). AI technology has the potential to predict whether patients with PTC harbor BRAF p.V600E mutations by analyzing and identifying the morphological features of cells (47). Nishikaw et al. generated a morphological analysis dataset using deep learning, constructed 72 whole-slide images, and extracted six types of nuclear features. This study successfully established a predictive model for identifying RET fusions, achieving an AUC of 0.801 (22). Additionally, integrative multiomics analyses—such as combining spatial proteomics, genomics, immunohistochemistry, and metabolomics—with the application of AI and machine learning methods can reveal complex relations and interactions among various molecular components, providing a more comprehensive biological landscape for pathological thyroid diagnosis and addressing current diagnostic challenges (48). Matrix-assisted laser desorption/ionization mass spectrometry imaging and desorption electrospray ionization mass spectrometry imaging enhance the diagnostic performance of FNA by effectively distinguishing between benign and malignant cell regions, serving as supplementary tools for diagnosing uncertain characteristics of thyroid nodules (15, 49) (**Table 1**).

**Table 1:** AI studies for thyroid disease diagnosis.

Study	Modality	Model type	AI task	Validation cohort & type	Dataset	Model validation performance	Limitations
Guan, Q., et al. (12)	Cytological images	VGG-16 DCNN	Classification	Single-center Retrospective Internal validation	279 cytological images of thyroid nodules	Spec 94.9% Sens 100.0% Acc 97.7% PPV 95.8% F1 score 0.98	This is a pilot study, they have only included a few typical cytological images.
Zhao et al. (14)	Cytological images	ResNeSt	Classification	Single-center Retrospective Internal validation	Training dataset 1,330 samples Test dataset 173 samples	Spec 88.5% Sens 95.8% Acc 92.5% F1 score 0.93	ResNeSt model could ‘t distinguish some misdiagnosed fragmented image because of VGG-16 made the diagnosis based on the size and staining of the nucleus, thus a combination of different models may provide better categorization.
Capitoli, G., et al (15)	Cytological images	MALDI-MSI model	Classification	Single-center Prospective External validation	207 patients	Spec 82.9% Sens 43.1% Acc 67.7% PPV 60.9% F1 score 0.50	Predictive values depend on malignancy prevalence. Narrow malignant case spectrum may impact model’s carcinoma signature recognition.
Li. et al. (18)	US	S-Detect	Classification	Single-center Prospective External validation	236 patients	AUC 0.753 Spec 56.0% Sens 95.0% Acc 84.0%	High proportion of malignant thyroid nodules. Part of benign group were based on US-guided biopsy results. There were no pathological results.This was a single-center study.
Swan, K.Z.,et al (19)	US	AIBx	Risk stratification	Single-center Retrospective External validation	209 patients	AUC 0.61 Spec 44.2% Sens 78.4% PPV 25.8% F1 score 0.39	This was a retrospective study, and only one image of each nodule was available. Image obtained only in the transversal plane.
Ha, E.J., et al. (20)	US	AI-Thyroid	Classification	Multicenter Retrospective External validation	Training 6163 patients Test set 1 4820 patients Test set 2 2367 patients	Test1 Test2 AUC 0.922 0.938 Spec 81.5% 81.6% Sens 87.0% 89.9%	Biases in data selection and misleading benign from malignant nodules. AI-Thyroid outcomes not compared to physicians’. Further studies on “nondiagnostic” or “indeterminate” nodules needed.
Nishikawa,T.,et al. (22)	Hematoxylin and eosin staining slides	Four convolutionalneural networks	Gene prediction	Multicenter Retrospective	72 samples of classical papillary thyroid carcinoma.	AUC: RET fusions 0.801; BRAFp.V600E mutation 0.638	The sample size for RET fusion cases and the training dataset for nuclear feature detection were small. Only BRAFV600E-negative cases were tested for RET fusions.
Peng, S., et al. (27)	US	ThyNet	Classification	Multicenter Prospective External validation	Training 8339 patients. Total test 2775 patients	AUC 0.823 Spec 89.1% Sens 94.9%	The prevalence diversity could impact PPV and NPV, reducing results’ generalizability. Radiologists’ performance may be underestimated.
Yu, J., et al. (31)	US	TLR model	Metastasis prediction	Multicenter Prospective External validation	The first two datasets 3172 patients The third dataset 1691 patients	Test1 Test2 AUC 0.93 0.93 Spec 89.0% 75.0% Sens 83.0% 95.0% Acc 86.0% 84.0% PPV 92.0% 74.0% F1 score 0.87 0.83	/
Kwon, M.R., et al. (32)	US	Logistic regression SVM. Random forest using 5-fold CV.	Gene prediction	Single-center Retrospective Internal validation	96 thyroid nodules	AUC 0.65 Spec 61.8% Sens 66.8% Acc 64.3%	This was a retrospective study from a single institution with small datasets. The lack of external validation data.
Zhu, Y., et al. (33)	US	CUS+UE+CEUS	Prediction primary cancer sites	Single-center Retrospective Internal validation	280 cancer patients	AUC 0.838 Spec 94.5% Sens 59.7% Acc 87.3% PPV 74.5% F1 score 0.66	The study was subject to selection bias, using the same scanner for multimodal ultrasound images and focusing on one region of interest from B-mode, color Doppler, and elastography images for model building.
Gao, Y., et al. (34)	US	An integrated model with DL, radiomics, and clinical imaging features.	Metastasis prediction	Single-center Retrospective Internal validation	613 patients	AUC 0.841 Spec 81.8% Sens 72.4% Acc 77.1% PPV 79.7% F1 score 0.76	US images of central LNs were not included in the analysis. The interpretability of features learned by the DL and radiomics model remains limited. The limited amount of data utilized.
Lv, X., et al. (35)	US and frozen section	The clinical model, radiomics model and nomogram.	Metastasis prediction	Multicenter Retrospective Internal validation	208 patients	AUC 0.803 Spec 53.6% Sens 100%	Selection bias. The sample size was small.Radiomics feature variability from equipment and settings is unexamined. Did not divide cervical LNS into central and lateral.
Park, V.Y., et al. (36)	US	A radiomics signature (Rad-score) based on thyroid.	Predicting disease-free survival	Single-center Retrospective	768 patients.	C-index 0.777 Rad-score 3.087	The retrospective nature of its data collection and the relatively small sample size.
Liu Y., et al. (37)	US	AI TI-RADS	Classification	Multicenter Retrospective External validation	1859 patients.	AUC 0.762 Spec 70.2% Sens 82.2% Acc 73.3% PPV49.0% F1 score 0.61	Selection bias. The study’s composite reference standard may contain potential errors. The sample does not accurately represent real-world thyroid nodule types. Agreement was assessed only between two readers.
Wang, C., et al. (38)	CT	AI-based CLNM prediction system.	Metastasis prediction	Multicenter Retrospective External validation	Development set 423 patients Internal test set 182 patients. External test set 66 patients.	AUC 0.81 Spec 92.0% Sens 62.0% Acc 73.0% PPV 93.0% F1 score 0.74	Samples were obtained only in China. Manual segmentations were performed by a radiologist. Tumor diameters<0.5 cm were not included.
Wei, R., et al. (39)	MRI	A radiomics predictive model.	Metastasis prediction	Single-center Retrospective Internal validation	132 patients	Spec 93.4% Sens 89.5% Acc 91.7% PPV91.1%	The sample size was small and not externally validated. Lesions varied significantly between ETE and non-ETE groups. Tumors < 5 mm were excluded.
Chan WK, et al. (43)	US	InceptionV3^1^ ResNet101^2^ VGG19^3^	Classification	Single-center Retrospective Internal validation	812 patients	AUC 0.82^1^ 0.83^2^ 0.83^3^ Spec% 76.9^1^ 81.4^2^ 83.7^3^ Sens%76.0^1^ 72.5^2^66.2^3^ Acc % 76.5^1^77.6^2^76.1^3^ PPV % 71.8^1^75.1^2^75.8^3^ F1score0.74^1^0.74^2^0.71^3^	Selection bias. Collecting malignant samples is time-consuming. Test set has few cases, lacks tumor size info. Rarer malignant forms excluded. Diagnostic power for multinodular goiters unclear. Ultrasound algorithm differences impact training and classification.
Wang CW, et al. (47)	Cytological images	DLframework	Gene prediction	Single-center Retrospective Internal validation	118 whole slide images	Spec 71.0% Sens 91.0% Acc 87.0% PPV 94.0% F1 score 0.92	Selection bias. The retrospective nature of its data collection and the relatively small sample size.
DeHoog, R.J., et al. (49)	Tissue samples	DESI-MSI Test1 Benign *vs*. PTC model Test2 Benign *vs*. FTC model	Classification	Multicenter Retrospective Internal validation	206 frozen human thyroid tissue samples	Test1 Test2 Spec 91.0% 88.0% Sens 96.0%100.0% PPV 88.0%20.0% F1 score 0.92 0.33	Access to patient and clinical information is limited. The study had one FNA FTC sample. DESI-MS imaging requires frozen FNA samples for stability.Imaging experiments take a few hours.

AUC, Area under the curve; Acc, Accuray;CV, cross-validation; CLNM, Central lymph node metastasis; DCNN, Deep convolutional neural network; DL, Deep learning; DESI-MS, Desorption electrospray ionization mass spectrometry; ETE, extrathyroidal extension; LNs, Lymph nodes; MALDI-MSI, Matrix-assisted laser desorption/ionization mass spectrometry imaging; NPV, Negative predictive value; PPV, Positive predictive value; Sens, sensitivity; Spec, specificity; SVM, Support vector machine; UE, Ultrasound elastography; US, Ultrasound.

### 3.2 Therapeutic applications

#### 3.2.1 Surgical decision-making

Radiomic models can analyze risk stratification, predict the invasiveness of thyroid cancer and lymph node metastasis, and guide surgical decisions regarding preventive lymph node dissection (50). One study used of mind maps and iterative decision trees to develop a guideline-based clinical decision support system for routine surgical practice. The concordance between clinical decision support system recommendations and actual treatment decisions in real-world clinical settings was 78.9% (51).

#### 3.2.2 Targeted therapy guidance

Initially, the concept of personalized treatment relied primarily on clinical experience and pathological analysis and lacked data-driven approaches. Advancements in AI have facilitated a gradual shift toward data-driven personalized treatment. With the development of genomics and bioinformatics, researchers have begun using AI to analyze patient genetic information to predict disease risk and treatment responses (52). Early studies focused on the genetic mutation analysis of patients with thyroid cancer to identify biomarkers associated with treatment sensitivity (21, 53). The ResNet152-based DTLR model demonstrated significant value in identifying BRAF p.V600E mutations in patients with PTC using ultrasound images (54).Combination therapy with dabrafenib and trametinib is currently the standard treatment for patients with the BRAF p.V600E mutation. Machine learning approaches have contributed to the identification of biological pathways involved in cancer drug responses. For example, machine learning methods identified Rac1/cytoskeleton signaling transduction as the most significant driver of resistance to BRAF inhibitors (55). AI-assisted virtual screening identified Kir5.1 as a druggable target through the molecular docking of 200,000 compounds. Additionally, 10 potent compounds that interact with Kir5.1 were successfully identified using AI-assisted virtual screening (24) (**Table 2**).

**Table 2:** AI studies for therapeutic applications.

Study	Modality	Model type	AI task	Validation cohort & type	Dataset	Model validation performance	Limitations
Yang, X., et al. (24)	Gene Expression Omnibus database	.A.I. system AlphaFold AutoDock Vinav.1.2.0	Gene prediction	Single-center Prospective External validation	70 pairs of thyroid tumor and paratumor tissues	Kir5.1 is a potential therapeutic target for thyroid cancer. Identified genes and developed Kir5.1 interaction compounds.	Sample capacity was insufficient, specifically owing to the lack of DeTC and Anaplastic thyroid carcinoma specimens.Can not reflect thelocation or distribution of DEGs in cells.
Fan, F., et al. (50)	US	Combined model	Metastasis prediction	Single-center Retrospective Internal validation	211 patients.	AUC 0.901 Spec 86.7% Sens 82.4% Acc 84.4%	Their analysis had a small sample size, focusing on static grayscale ultrasound images of LNs without multimodal image integration. LN size classification was not detailed.
Yu HW., et al. (51)	Clinical data	Clinical knowledge models	Clinical decision support system	Single-center Retrospective	.483 patients	CDSS recommendations and clinical treatment concordance was 78.9%.	To implement a CDSS, a large-scale study is required. Limited cases prevent assessing some IDT paths with SNUBH data. The concordance rate is 78.9%, which is low.
Liu, Y., et al. (53)	Cancer genome atlas database	The Pathomics model	Gene prediction	Multicenter Retrospective External validation	401 cases	AUC 0.769	Variability in public databases. The majority of cases were stage I/II and of the traditional subtype, potentially biasing the data analysis.
Wu F., et al. (54)	US	The ResNet152-based DTLR model	Gene prediction	Single-center Retrospective Internal validation	738 patients	AUC 0.833 Spec 81.7% Sens 76.2% Acc 80.6% PPV 49.2% F1 score 0.60	Although itincluded numerous samples, there is still the potential for study enlargement. Selection bias. This study employed manual tumor segmentation, noting the variability among individuals.

Acc, Accuray; AUC, Area under the curve; CDSS, Clinical decision support system; DEGs, differentially expressed genes; DeTC, Dedifferentiated thyroid cancers; DTLR, deep transfer learning radiomics; IDT,Iterative decision tree; LNs,Lymph nodes; PPV,Positive predictive value; Sens, sensitivity; SNUBH, Seoul National University Bundang Hospital; Spec, specificity.

### 3.3 Prognostic monitoring

AI not only holds significant promise in the diagnosis and personalized treatment of patients with thyroid diseases but also plays an increasingly important role in patient monitoring and follow-up, particularly in remote monitoring, prognostic assessment, and risk management.

#### 3.3.1 Recurrence prediction

With the maturation of deep and machine learning algorithms, the application of AI in the personalized treatment of thyroid diseases has continued to expand. Researchers have begun integrating clinical data, imaging features, and biomarkers to develop complex predictive models. These models not only assist physicians in formulating individualized treatment plans but also evaluate patients’ responses to various therapies. Zhang et al, integrated radiomic features, mutated genes, and clinical characteristics to construct a nomogram model. The study found that this model significantly enhanced the predictive efficacy of radiomic features for lymph node metastasis improving accuracy from 71.5% to 87.0% (23).

In the context of prognostic assessment and risk management, AI aids in analyzing long-term data to evaluate the risk of recurrence, particularly during the follow-up of patients with thyroid cancer. Timely interventions can be initiated by the early detection of abnormal signals. For instance, one study analyzed the prognostic significance of clinical and pathological factors in 1,040 patients with PTC, including the number of metastatic lymph nodes and lymph node ratio. Researchers attempted to construct a disease recurrence prediction model using machine learning techniques and compared the accuracy of five machine learning models. The decision tree model exhibited the highest accuracy at 95%, while the combination of Light Gradient Boosting Machine and stacking models showed an accuracy of 93% (25). In another study involving 554 patients with PTC, researchers used radiomic features in combination with significant clinical and pathological characteristics to construct a nomogram. The results demonstrated that the combined nomogram showed strong concordance with actual recurrence events and yielded a net benefit superior to that of traditional clinical models across most thresholds (26).

#### 3.3.2 Remote monitoring

With ongoing technological advancements, the application of AI in remote monitoring has steadily increased. Using smartphones and wearable devices, patients’ physiological parameters and symptoms can be collected and transmitted to healthcare teams in real time (56, 57). AI systems can analyze these data to promptly identify potential complications and recurrence risks, thereby providing physicians with real-time decision-making support. This form of remote monitoring not only enhances patients’ self-management capabilities but also reduces the need for frequent clinic visits (**Table 3**).

**Table 3:** AI studies for prognostic monitoring.

Study	Modality	Model type	AI task	Validation cohort & type	Dataset	Model validation performance	Limitations
Zhang, R., et al. (23)	Samples obtained by fine-needle aspiration	Multi-feature integration nomogram model for LNM prediction	Metastasis prediction	Single-center Prospective Internal validation	182 thyroid nodule samples.	AUC 0.603	**/**
Park, Y.M. and B.J. Lee (25).	US	The decision tree, RF, XGBoost, and LightGBM, and Stacking models.	Recurrence prediction	Single-center Retrospective Internal validation	1040 patients	Sens18% Acc 95.0% PPV 66.0% F1 score 0.28	Need more patient data. This was a retrospective study conducted at a single institution and there were selection bias.
Zhou, B., et al. (26)	US	Clinical model, radiomics signature and combined nomogram.	Recurrence prediction	/	554 patients Training cohort 388. Validation cohort166	AUC 0.885 Spec 86.4% Sens 75.0% Acc 86.1%	This was a retrospective study conducted atone center and there were certain potential biases. Small sample size and the short follow-up time.
Chen, J., et al. (56)	US	Flexible and stretchable ultrasound transducer	Wearable devices.	/	/	With large tensile strains(≥110%), high flexibility (R ≥ 1.4 mm), and lightweight (≤1.58 g)to meet the needs of wearable devices.	Ignoring array elements during large curvature surface conformation can lead to phase compensation errors due to discrepancies between actual and defined positions.
Kim, K.H., et al. (57)	Wearable device	Heart rate monitored by a wearable device	Wearable devices.	Single-center Prospective	44 patients	/	Results may not apply to those new to smart devices. Gender ratio varied between groups, with a small, imbalanced sample size. Study’s validity for early or mild hypothyroidism is uncertain.

Acc, Accuray; AUC, Area under the curve; LNM, Lymph node metastasis; PPV, Positive predictive value; RF, Random forest;Sens, sensitivity; Spec, specificity.

## 4 Conclusion and outlook

AI has demonstrated significant potential in the detection and follow-up of patients with thyroid diseases, particularly in imaging analysis, prediction of invasiveness and metastasis, and prognostic assessment. Through deep learning and machine learning techniques, AI has not only improved the accuracy of differentiating between benign and malignant thyroid nodules but also integrated multiple data sources to monitor patient health and identify potential risks in a timely manner. Despite the promising prospects of AI in thyroid disease management, critical challenges persist regarding data privacy, model interpretability, and clinical applicability. This study had three fundamental limitations:

First, the generalization capacity of AI models is profoundly affected by dataset homogeneity. Existing studies predominantly relied on single-center, hospital-based cohorts (28 of 30 studies, 93%), which differ in thyroid cancer prevalence compared with the general population, thereby compromising external validity. Notably, 83% of the models (25 of 30) were trained on Asian datasets, raising concerns about their efficacy across diverse ethnic and geographic populations. Furthermore, the inadequate representation of pathological subtypes—with 90% of studies focusing on classical PTC—has resulted in diagnostic inequity for patients with FTC and other rare subtypes. This limitation contributes to degraded algorithmic performance across institutions, imaging devices, and multiethnic cohorts.

Second, the “black-box” nature of AI models remains a critical barrier to clinical adoption. Although interpretability tools, such as SHapley Additive exPlanations and Local Interpretable Model-agnostic Explanations (58–60), have been partially implemented, current systems fail to transparently elucidate decision-making pathways—particularly the relative contributions of key morphological features, such as microcalcifications versus vascular patterns. This opacity complicates the clinical validation of misdiagnoses, including the erroneous classification of Hashimoto’s thyroiditis as malignancy (15).

Third, these two systemic disconnects hinder real-world application. Algorithmic development remains poorly integrated with clinical workflows, as exemplified by models, such as those developed by Peng et al. (27), which lack compatibility with Picture Archiving and Communication Systems. Concurrently, the absence of ethical and legal frameworks—addressing liability attribution for AI misdiagnoses and informed consent for predictive genomic models—creates regulatory ambiguities (61).

Future research should prioritize these three directions. Cross-modal data-fusion architectures must integrate ultrasound, pathomics, and multiomics data to develop interpretable multitask learning frameworks. Algorithmic improvements are urgently required to enhance predictive fairness in heterogeneous thyroid nodule populations. The seamless integration of AI tools into clinical workflows necessitates the establishment of rapid implementation pipelines. Additionally, prospective randomized controlled trials are imperative to quantify the real-world impact of AI systems on healthcare costs—such as reductions in FNA rates—and patient outcomes, including 5-year survival rates. Addressing these priorities will bridge the gap between AI innovation and equitable, ethically grounded clinical practice.
